# Long-Term Effects of Intermittent IL-2 in HIV Infection: Extended Follow-Up of the INSIGHT STALWART Study

**DOI:** 10.1371/journal.pone.0047506

**Published:** 2012-10-17

**Authors:** Norman Markowitz, Gustavo Lopardo, Deborah Wentworth, Daniela Gey, Abdel Babiker, Lawrence Fox, Jorge Tavel, STALWART Study Group

**Affiliations:** 1 Henry Ford Hospital, Detroit, Michigan, United States of America; 2 Fundación Centro de Estudios Infectológicos, Buenos Aires, Argentina; 3 Division of Biostatistics, University of Minnesota, Minneapolis, Minnesota, United States of America; 4 Copenhagen HIV Programme, University of Copenhagen, Copenhagen, Denmark; 5 Medical Research Council, London, England, United Kingdom; 6 National Institute of Allergy and Infectious Diseases, Bethesda, Maryland, United States of America; University of Cape Town, South Africa

## Abstract

**Background:**

The Study of Aldesleukin with and without Antiretroviral Therapy (STALWART) was designed to evaluate whether intermittent IL-2 alone or with peri-cycle ART increased CD4+ cell counts (and so delayed initiation of ART) in HIV infected individuals having ≥300 CD4+ cells/mm^3^ compared to untreated controls. When the results of two large clinical trials, ESPRIT and SILCAAT, showed no clinical benefit from IL-2 therapy, IL-2 administration was halted in STALWART. Because IL-2 recipients in STALWART experienced a greater number of opportunistic disease (OD) or death and adverse events (AEs), participants were asked to consent to an extended follow-up phase in order to assess persistence of IL-2 effects.

**Methodology:**

Participants in this study were followed for clinical events and AEs every 4 months for 24 months. Unadjusted Cox proportional hazards models were used to summarize death, death or first OD event, and first grade 3 or 4 AE.

**Principal Findings:**

A total of 267 persons were enrolled in STALWART (176 randomized to the IL-2 arms and 91 to the no therapy arm); 142 individuals in the IL-2 group and 80 controls agreed to enter the extended follow-up study. Initiation of continuous ART was delayed in the IL-2 groups, but once started, resulted in similar CD4+ cell and viral load responses compared to controls. The hazard ratios (95% CI) for IL-2 versus control during the extension phase for death or OD, grade 3 or 4 AE, and grade 4 AE were 1.45 (0.38, 5.45), 0.43 (0.24, 1.63) and 0.20 (0.04, 1.03), respectively. The hazard ratios for the AE outcomes were significantly lower during the extension than during the main study.

**Conclusions:**

Adverse events associated with IL-2 cycling did not persist upon discontinuation of IL-2. The use of IL-2 did not impact the subsequent response to initiation of cART.

## Introduction

STALWART [1] was designed to evaluate the safety, immunologic, and virologic effects of intermittent interleukin 2 (IL-2) in asymptomatic HIV-infected persons not receiving antiretroviral therapy (ART) and having a CD4+ cell count ≥300 cells/mm^3^. The study tested the hypothesis that IL-2 with or without peri-cycle ART would maintain or increase CD4+ cell counts compared to controls receiving neither IL-2 nor ART. The maintenance of higher CD4+ values with IL-2 was hypothesized to permit the safe deferral of continuous ART (cART) for a longer period of time compared to controls. Overall, 267 individuals were randomized: 89 to receive IL-2, alone; 87 to receive IL-2 and peri-cycle ART; and 91 untreated controls. Most (78.7%) were ART naïve; the remainder, according to protocol, had not taken any ART within the preceding year. When the results of ESPRIT and SILCAAT showed that IL-2 combined with continuous ART conferred excess toxicity without clinical benefit [2] further IL-2 administration was halted in STALWART, and safety and efficacy data were reported [1]. Although IL-2 recipients in STALWART experienced a significant increase in CD4+ cell count (the change from baseline to week 32 was approximately 130 cells/mm^3^ higher in IL-2 recipients compared to controls), grade 3 or 4 adverse events were also significantly more common among those given IL-2. Moreover, a trend was observed for an increased risk of opportunistic diseases or death among IL-2 recipients compared to untreated controls. Thus, the IL-2 induced CD4+ cell count elevation did not appear to accurately represent an improvement in immune function.

Compared to controls, cART was less likely to have been started among IL-2 recipients during the study period. This was likely attributable to the higher CD4+ counts observed among those receiving IL-2, which, when taken at face value, would have less frequently led to the initiation of ART. This “delay” in the initiation of antiretrovirals may have accounted, at least in part, for the increased opportunistic events observed among those who received IL-2; notably, no STALWART participant who experienced an opportunistic disease was on continuous ART when the event occurred. At the time of STALWART closure, the hazard ratios (IL-2 groups combined versus controls) for opportunistic disease (OD) or death and for moderate or severe adverse events (AEs) were 5.8 (0.76–45.1, p = 0.09) and 2.9 (1.4–6.0, p = 0.004). Because the persistence of the IL-2 induced CD4+ cell quantitative versus qualitative discordance was unknown as well as the long-term toxicity, participants were asked to consent to an additional two years of unblinded clinical observation in an extended follow-up phase of STALWART.

## Methods

### Ethics

The STALWART study was approved by the institutional review board (IRB) or institutional ethics committee (IEC) of each clinical site and of the University of Minnesota, and informed consent was obtained from all participants. A letter of amendment to the protocol was issued in April 2009 continuing unblinded safety assessments for an additional two years beyond the planned follow-up. Re-consent was obtained from patients if required by that individual IRB/IEC.

### Design

The design, methods, and results of the STALWART trial have been previously published [1]. During the extended follow-up phase, patients were seen at four-month intervals for study visits where information on CD4+ cell count and HIV-RNA was collected. The following clinical events were reported as they occurred: deaths, opportunistic disease (OD) events (AIDS-defining events, bacterial pneumonia, and salmonella) and adverse events of grade 3 or 4 severity, as assessed by site investigators using a standard National Institute of Allergy and Infectious Diseases (NIAID) Division of AIDS toxicity table.

### Statistical Methods

Because the primary focus of the extended follow-up was on adverse consequences of IL-2 administration, and because the original study showed no difference in the rates of AEs and ODs between the two IL-2 groups, key events are summarized for both IL-2 groups combined versus control. Significance testing for differences between treatment groups is performed using chi square and Kruskal-Wallis tests. Kaplan-Meier survival curves are used to summarize time to initiation of continuous ART from randomization to the end of the extended follow-up phase. This analysis is based on all patients randomized, and follow-up is censored at the end of the main study for those patients who did not participate in the extended follow-up. Cox proportional hazards models, stratified by geographic region (7 strata total), are used to summarize all-cause death, the combined endpoint of death or first OD event, and first grade 4 or grade 3/4 AE. These events are summarized for two follow-up periods: from randomization through 28 February 2009, the originally planned end of study; and from 1 March 2009 to 28 February 2011, the extended follow-up phase. An expanded model with an interaction term between treatment indicator and time period was used to assess whether the hazard ratios (HR) differed between the two time periods. Rates are shown per 100-person months follow-up.

Other analyses, shown for the 3 treatment groups, are restricted to the patients who participated in the extended follow-up. During this phase, follow-up visits continued every four months according to a visit schedule that was generated at the time of randomization. Therefore, for analyses of the extended follow-up phase, visit windows were re-defined at four-month intervals (±2 months) following February 28, 2009, and visit-specific measurements were imputed from data available within that visit window and closest to the anniversary date.

Analyses of CD4+ cell count following initiation of cART were performed using a mixed longitudinal regression model with random patient effects and unstructured covariance matrix. These models were adjusted for age, gender, baseline CD4+ cell count and HIV-RNA, and last measured CD4+ cell count and HIV-RNA prior to ART initiation. For this analysis, CD4+ cell counts after ART initiation are ascribed to follow-up visits in a method similar to that described in the previous paragraph.

## Results

### Study Population

**Figure 1:**
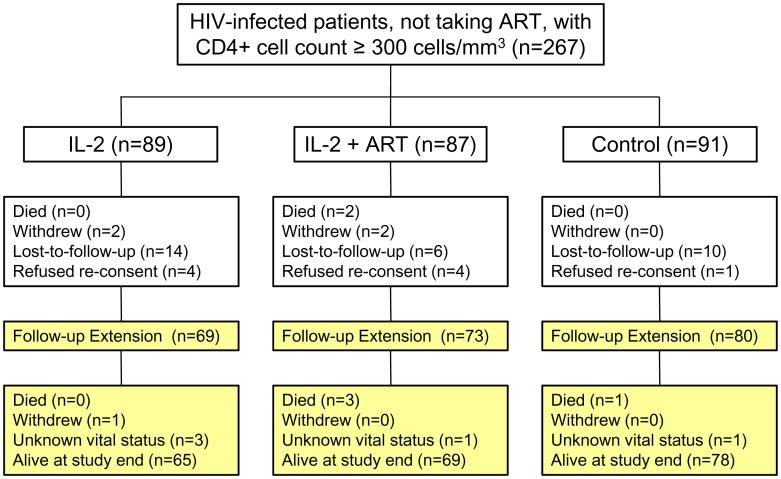
Study design and CONSORT flow diagram.

The extended follow-up phase of STALWART began on 1 March 2009 and continued to a common closing date of 28 February 2011. Among the 267 persons who entered STALWART, 222 (83.1%) entered the extended follow-up study (Figure 1): 69/89 recipients of IL-2; 73/87 recipients of IL-2+ peri-cycle ART (IL2+ART); and 80/91 controls. Overall, 80.7% of the patients assigned IL-2 compared to 87.9% of the control group participated in extended follow-up (p = 0.14). Across treatment groups, based on the last available measurement prior to beginning the extension phase, individuals who participated in extended follow-up were more likely to have undetectable HIV-RNA levels (26.6% versus 11.6%, p = 0.04). No other characteristics examined were determinates of participation in the extension phase.

The baseline (as randomized into STALWART) characteristics were similar across treatment groups for patients participating in extended follow-up (Table 1). At the time the extension phase began, 24.6% of IL-2 recipients (30.4% of those randomized to IL-2 alone and 19.2% of those randomized to IL-2+ART) had already started cART, compared to 43.8% of controls (p = 0.003). As a likely consequence of these differences, the median HIV-RNA was higher (13,895 copies/ml versus 3,045, p = 0.001) and percent with HIV-RNA ≤400 copies/ml was lower (21.1 versus 36.3, p = 0.01) for IL-2 recipients. The median CD4+ cell counts were 474 cells/mm^3^ among IL-2 recipients and 412 cells/mm^3^ among controls (p = 0.06). There were no differences in HIV-RNA or CD4 across treatment groups after stratification by whether patients had started cART (data not shown).

**Table 1:** Characteristics of STALWART participants at study entry and at the beginning of the extension phase.

	Study Entry		Beginning of Extension Phase	
	IL-2 (n = 176)	Control (n = 91)	IL-2 (n = 142)	Control (n = 80)
Age at baseline, median years	35	36	36	36
Gender, % female	17.0	17.6	17.6	17.5
Race, %				
Asian	25.6	24.2	26.1	26.3
Black	4.5	8.8	2.8	8.8
White/other	69.9	67.0	71.1	65.0
Likely mode of HIV infection (%)*				
Sexual, same sex	58.5	61.5	60.6	63.8
Sexual, opposite sex	39.8	37.4	39.4	35.0
Injection drug use	3.4	0.0	2.8	0.0
Other or unknown	3.4	2.2	2.1	2.5
Taking ART (%)	0.0	0.0	24.6	43.8
CD4+ cell count (median)	407	432	474	412
HIV-RNA (median copies/ml)	22,360	24,328	13,895	3,045
HIV-RNA ≤400 copies/ml (%)	3.4	4.4	21.1	36.3

* more than one may apply.

### Extended Follow-Up

#### Initiation of continuous ART

**Figure 2:**
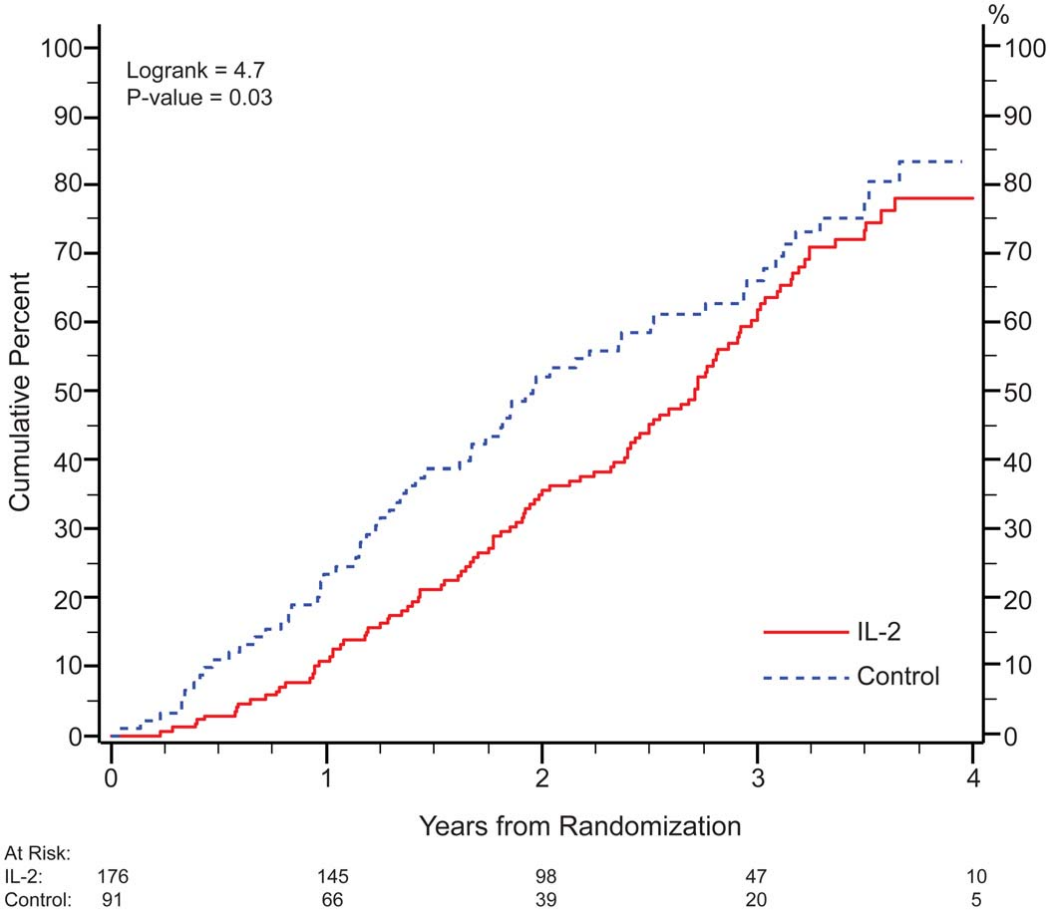
Time to initiation of continuous ART.

Upon being informed of results of the ESPRIT and SILCCAT trials, the STALWART protocol team, in recognition that the CD4+ cell counts might not reliably represent immune function, had recommended the initiation of cART for all IL-2 recipients. However, the decision to initiate ART was made by investigators and study participants on an individual basis. As seen in Figure 2, patients in the IL-2 groups initiated cART significantly later following initial randomization than those in the control group (p = 0.03). The CD4+ cell count at which ART was started was similar for both IL-2 recipients and controls: the median (IQR) CD4+ cell count proximal to the initiation of ART for the IL-2 group was 300 (230, 381) cells/mm3 versus 303 (232, 353) cells/mm3 for untreated controls. By the end of extended follow-up, 71.8% of the IL-2 group vs 78.8% of the control group had started cART.

#### CD4+ cells

**Figure 3:**
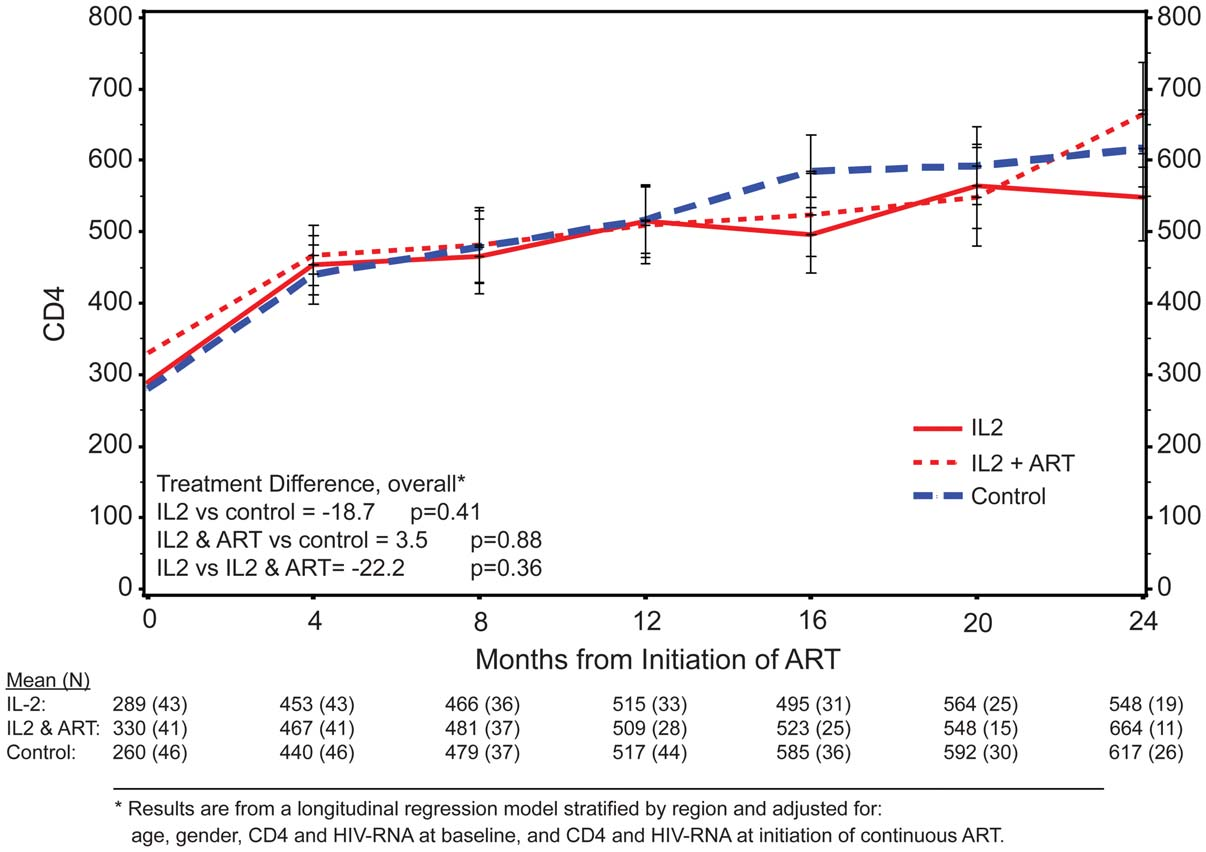
CD4+ cell count response after initiating continous ART.

At month 24 of extended follow-up, a month 24 CD4+ count was available for 48 recipients of IL-2 alone, 47 recipients of IL-2+ART, and 56 controls. For those given IL-2 alone and IL-2+ART, respectively, the mean (mean change from baseline) CD4+ cell counts were 496 cells/mm3 (+69.6 cells/mm3) and 512 cells/mm3 (+42.6 cells/mm3), while the mean CD4+ cell count (mean change from baseline) among controls was 559 cells/mm3 (+106.6 cells/mm3). Because of the delay in starting cART in the IL-2 groups, CD4+ cell counts were also analyzed from the time of initiation of cART. The adjusted CD4+ cell count response to cART was similar across all groups (Figure 3). Once started, the CD4+ cell response to cART appeared to have been unaffected by prior IL-2 or peri-cycle ART when compared to controls.

#### HIV RNA

**Figure 4:**
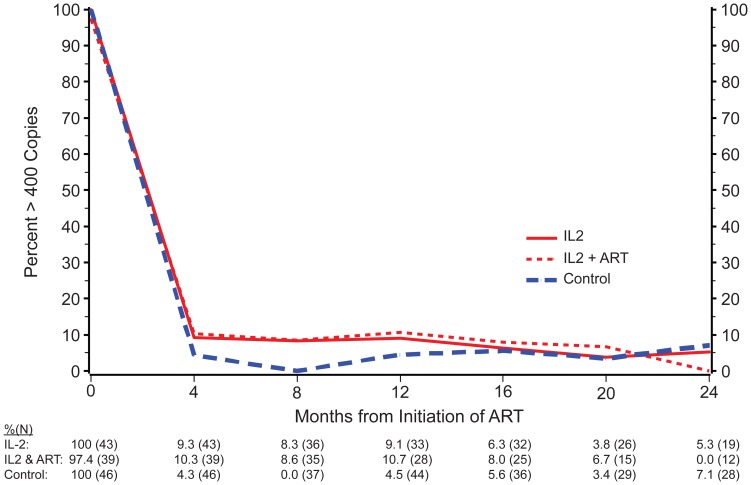
HIV-RNA after initiating continuous ART.

At the end of the extended follow-up, 49 IL-2 recipients, 48 IL-2+ART recipients, 57 controls had an available month 24 viral load. Between 69% and 74% of all treatment groups had HIV RNA levels ≤400 copies/ml. When treatment groups were compared following the initiation of cART, 90.7% of IL-2 recipients and 89.7% of those given IL-2+ ART had a viral load of ≤400 copies by month 4 after initiation of cART, compared to 95.7% in the control group (p = 0.55). Compared to controls, neither IL-2 nor peri-cycle ART appeared to impact the subsequent viral load response to cART (Figure 4).

#### Adverse events

The numbers of patients experiencing grade 3 and 4 adverse events are shown in Table 2 for the two study periods. While IL-2 recipients were at higher risk of experiencing a grade 3 or 4 event during the main study, the hazard ratio appeared to favor the IL-2 groups during the extension (HR = 0.63, 95% CI = 0.24–1.63, p = 0.34). The p-value for treatment group by time period interaction (a comparison of the HRs from the two time periods) was 0.01, indicating that the HR significantly declined from the first period to the second. Findings are similar when the analysis is restricted to grade 4 AEs (HR = 0.20, 95% CI = 0.04, 1.03, p = 0.05; interaction p-value = 0.04).

**Table 2:** Hazard Ratios for Key Events by Study Phase.

	IL-2		Control			
Event Type	No.	Rate	No.	Rate	HR (95% CI)	p-value
Grade 3 or 4 AE						
Main study	41	1.55	9	0.54	2.93 (1.42,6.04)	.004
Extension	9	0.28	8	0.43	0.63 (0.24,1.63)	.34
					Interactionp = 0.01	
Grade 4 AE						
Main study	11	0.34	3	0.17	2.31 (0.64,8.39)	.20
Extension	2	0.06	5	0.27	0.20 (0.04,1.03)	.05
					Interactionp = 0.04	
Death						
Main study	2	0.06	0	0.00		
Extension	3	0.09	1	0.05	1.63 (0.17,15.71)	.67
					Interactionp = 1.0	
Death or OD						
Main study	12	0.37	2*	0.11	2.94 (0.65,13.25)	.16
Extension	8	0.25	3	0.16	1.45 (0.38,5.45)	.59
					Interactionp = 0.31	

Table gives number of patients experiencing an event during the indicated study period and rate per 100 patient months follow-up. HR = hazard ratio (IL-2 versus control) from a proportional hazards model stratified by region. *Main study* refers to the period from randomization to 28 February 2009, the original planned end of the study (patients at risk: 176 IL-2, 91 control). *Extension* refers to the extended follow-up phase, from 1 March 2009 through 28 February 2011 (patients at risk: 142 IL-2, 80 control). Interaction p-values shown are for treatment group by study phase interaction.

* This reflects one additional OD event, in the control group, that was reported after the analysis and publication of the original study data.

#### Opportunistic disease and death

Of the 222 individuals who entered the extended follow-up phase of STALWART, 212 (95.5%) were known to be alive at the end of the study. Results for death and the combination endpoint of death or first OD event are displayed for the two phases of the trial in Table 2. Four persons (3 IL-2 recipients and 1 control) died during the extension (HR = 1.63, 95% CI = 0.17–15.71, p = 0.67). Eleven patients (8 versus 3) died or experienced an opportunistic event; the hazard ratio for this outcome was 1.45 (95% CI = .38–5.45, p = 0.59), compared to 2.94 during the main study (p = 0.31 for difference between study periods).

## Discussion

The follow-up phase of STALWART was designed to assess the outcome of patients who received IL-2 alone or IL-2+ peri-cycle ART. Because there was a trend towards an excess of clinical events among IL-2 recipients and because these events occurred at relatively elevated CD4+ cell counts, it was important to determine if this trend (which did not attain statistical significance) persisted after the discontinuation of IL-2. The elevated risk of adverse events associated with IL-2 administration did not persist during the extended follow-up phase. While a trend toward more opportunistic events in the IL-2 groups was observed during the extension, this may be in part explained by the delay in starting ART in the treatment groups assigned to IL-2.

Despite its ability to increase CD4+ T cells, IL-2 with continuous ART has been shown to confer no clinical benefit or protection from opportunistic diseases or death compared to continuous ART alone, but was associated with an increased risk of adverse events [2]. STALWART, which was undertaken before the results of ESPRIT and SILCAAT were known and stopped once these results became available, likewise showed no clinical benefit of IL-2 with or without peri-cycle ART when compared to no treatment among individuals for whom, at the time, treatment was not clinically indicated [1]. Because of an apparent increase in the number of opportunistic diseases or death among those who received any IL-2 in STALWART, as well as their occurrence at relatively elevated proximal CD4+ cell counts, participants were asked to participate in a 2 year extended follow-up study. Overall approximately 83% of participants were followed in the observation phase, representative of the initial randomized STALWART population. Over the follow-up period, compared to controls, persons who received IL-2 at any time did not experience an increased rate of grade 3/4 clinical events or opportunistic diseases/death. The subsequent response to continuous ART, once started, did not appear to have been impacted by prior IL-2 with or without peri-cycle ART. This observation is important given the concern that peri-cycle ART might lead to drug resistance and predispose to treatment failure once cART has been initiated, although in the initial randomized study peri-cycle ART was not clearly associated with the emergence of drug resistance based on population sequencing [1].

Although SILCAAT and ESPRIT showed that IL-2 plus cART did not lead to a reduction in incidence of OD/Death compared to ART alone, there were significantly more AEs – particularly thromboembolic events – among IL-2 recipients. The reasons for the lack of clinical benefit of IL-2, despite its ability to markedly increase CD4+ cell count, has not been fully elucidated. There have been no demonstrable long-term increases of plasma HIV RNA or intracellular HIV DNA [1], [3]–[8]. However, it is likely that the polyclonal CD4+ increases with prolonged survival of central memory and naïve T-lymphocytes [9]–[13] observed do not result in immunologically relevant protection. Various hypotheses have been proposed for this observation, including functional impairment of the IL-2-induced CD4+ cells, negative modulatory effects from other CD4+ subsets such as CD4+CD45RO-CD25+ foxP3 expressing cells, or lack of impact on the GALT [14]–[16]. Although immune activation measured by T-cell turnover is reduced among IL-2 recipients [10], [11], [17], IL-2 has been shown to acutely increase inflammatory markers, including D-dimer [18]–[20], which may contribute to hypercoagulability and to the increased death rate among ESPRIT participants having the greatest IL-2-induced CD4+ cell expansions [21].

Although the findings in ANRS 119, which like STALWART included IL-2 only versus untreated control arms, suggested that IL-2 usage might safely allow deferral of ART, its sample size was smaller and it was not powered to detect differences in clinical events [22]. The STALWART study suggests that this deferral may come at a price. However, it is possible that other immunomodulatory agents, such as IL-7 may have a future role [23], [24].

In conclusion, STALWART demonstrated that IL-2 conferred no clinical benefit among asymptomatic HIV-infected persons not receiving antiretroviral therapy. While its administration led to a delay in the initiation of ART, this delay was associated with a trend towards a greater incidence of OD/Death and a statistically significant increase in AEs. Over the extended follow up, event rates in the IL-2 groups remained higher than controls, but differences were not statistically significant. Although the time from discontinuation of IL-2 to the reversal of its effects could not be measured in this extension study, the effects of IL-2, with or without peri-cycle ART, were not persistent and not associated with any lingering impact on the response to subsequent ART.
